# The Impact of Primer Design on Amplicon-Based Metagenomic Profiling Accuracy: Detailed Insights into Bifidobacterial Community Structure

**DOI:** 10.3390/microorganisms8010131

**Published:** 2020-01-17

**Authors:** Leonardo Mancabelli, Christian Milani, Gabriele Andrea Lugli, Federico Fontana, Francesca Turroni, Douwe van Sinderen, Marco Ventura

**Affiliations:** 1 Laboratory of Probiogenomics, Department of Chemistry, Life Sciences and Environmental Sustainability, University of Parma, 43124 Parma, Italy; leonardo.mancabelli@genprobio.com (L.M.); christian.milani@unipr.it (C.M.); gabrieleandrea.lugli@unipr.it (G.A.L.); federico.fontana4@studenti.unipr.it (F.F.); francesca.turroni@unipr.it (F.T.); 2 APC Microbiome Institute and School of Microbiology, Bioscience Institute, National University of Ireland, T12 YT20 Cork, Ireland; d.vansinderen@ucc.ie

**Keywords:** microbiota, 16S rRNA profiling, metagenomics, primer pairs, *Bifidobacterium*

## Abstract

Next Generation Sequencing (NGS) technologies have overcome the limitations of cultivation-dependent approaches and allowed detailed study of bacterial populations that inhabit the human body. The consortium of bacteria residing in the human intestinal tract, also known as the gut microbiota, impacts several physiological processes important for preservation of the health status of the host. The most widespread microbiota profiling method is based on amplification and sequencing of a variable portion of the 16S rRNA gene as a universal taxonomic marker among members of the Bacteria domain. Despite its popularity and obvious advantages, this 16S rRNA gene-based approach comes with some important limitations. In particular, the choice of the primer pair for amplification plays a major role in defining the accuracy of the reconstructed bacterial profiles. In the current study, we performed an in silico PCR using all currently described 16S rRNA gene-targeting primer pairs (PP) in order to assess their efficiency. Our results show that V3, V4, V5, and V6 were the optimal regions on which to design 16S rRNA metagenomic primers. In detail, PP39 (Probio_Uni/Probio_Rev), PP41 (341F/534R), and PP72 (970F/1050R) were the most suitable primer pairs with an amplification efficiency of >98.5%. Furthermore, the *Bifidobacterium* genus was examined as a test case for accurate evaluation of intra-genus performances at subspecies level. Intriguingly, the in silico analysis revealed that primer pair PP55 (527f/1406r) was unable to amplify the targeted region of any member of this bacterial genus, while several other primer pairs seem to rather inefficiently amplify the target region of the main bifidobacterial taxa. These results highlight that selection of a 16S rRNA gene-based PP should be done with utmost care in order to avoid biases in microbiota profiling results.

## 1. Introduction

During the last decade, a growing number of studies have described efforts to profile complex microbial communities, i.e., microbiota, hosted by various human body sites. Scientific evidence has highlighted that the human microbiota plays a major role in several physiological host activities that are essential for health maintenance [1,2]. Such in-depth studies of, in particular, the human gut microbiota has been facilitated by the development of high-throughput sequencing technologies, such as Roche 454, Ion Torrent, and Illumina which allow accurate profiling of bacterial populations without the need for their cultivation [1]. These next-generation approaches facilitated the birth of a research field called metagenomics by providing an efficient tool for taxonomic and functional profiling of non-cultivable bacteria, thereby allowing a comprehensive overview of complex bacterial communities.

Among metagenomic approaches, the 16S rRNA gene-based profiling method is currently the most commonly employed to catalogue components of the human microbiota. This methodology has many advantages, such as reproducible and technically easy procedure, high efficiency in the identification of bacterial phylogeny and taxonomy, accessible bioinformatic pipelines, and low cost, which rapidly led to its wide spread use [3]. Despite these advantages, 16S rRNA gene-based profiling analysis also comes with some important limitations, such as DNA extraction method and the annealing efficiency of the primers used for the amplification step [4], which can considerably influence the bacterial community profiling output. Moreover, the overall length of the 16S rRNA gene is falling outside the maximum read length achievable by the common high-throughput sequencing technologies, e.g., Illumina platforms, thus precluding a high taxonomic resolution of the generated microbial profiles of complex bacterial communities [5]. In this context, one of the most critical steps of this community profiling method is amplification of a specific hypervariable region of the 16S rRNA gene that is then sequenced and used for taxonomic reconstruction. In detail, the primer pair that is used to amplify this region from each taxonomic group present in the biological sample may be responsible for biases in the obtained results [6]. In fact, previous studies have shown conflicting reports regarding the composition of the gut microbiota caused by the use of different primers, which led to an under- or over-representation of specific components of the bacterial community in the human gut [7,8]. For example, several published studies have reported that the abundance of members of the genus *Bifidobacterium,* as determined by such culture-independent metagenomic studies, was likely to have been underestimated due to PCR primer biases [4,9,10]. In particular, several studies regarding the profiling of the infant gut microbiota, which used different metagenomics methodologies, have in some cases resulted in conflicting results, with certain discrepancies in the bifidobacterial relative abundance [4].

In the current study, we performed an in silico PCR analysis in order to assess the efficiency of 75 different primer pairs, which represent all currently described PCR primers designed for universal bacterial profiling analysis by amplification of a specific portion of the 16S rRNA gene. Furthermore, to gain insight into the relevance of intra-genus species-level performances, the efficiency of these 75 primer pairs was tested using a custom database encompassing commonly encountered bifidobacterial species.

## 2. Materials and Methods

### 2.1. Selection of Primer Pairs

A total of 75 primer pairs were included in this study. These primer pairs were selected from the online database probeBase (https://doi.org/10.1093/nar/gkv1232), which includes rRNA-targeting oligonucleotide primers and probes, and from publicly available and published metagenomic analyses obtained from 16S rRNA gene-based profiling (Figure S1 and Table S1). Primers capable of specifically amplifying Archaea and Eukaryotes have been excluded.

### 2.2. In Silico PCR

The performance of primer pairs employed in the study was evaluated through the web-tool TestPrime 1.0 [7]. The latter performs an in silico PCR using the SILVA database as a template, and provides the percentage of amplified sequences for each bacterial genus [7]. The TestPrime was based on the RefRN SILVA Database ssu-132, and a maximum of three mismatches was allowed [11].

### 2.3. 16S rRNA-Based Microbiota Analysis of Public Datasets

All datasets included in this meta-analysis were selected from publications based on comparative human gut microbiota studies. In detail, we collected 16S rRNA gene-based profiling datasets from six studies, three of which were using primer pairs with an efficiency <50%, i.e., PP5, PP14, and PP23, and three based on 16S rRNA profiling primers with a predicted amplification efficiency of >90%, i.e., PP39, PP45, and PP50 (Table S4). Therefore, datasets corresponding to 10 fecal samples obtained from healthy adult humans were randomly selected among those used in each study, for a total of 60 datasets. Additionally, we also randomly selected a total of 19 datasets belonging to two different studies assessing the fecal microbiota of healthy infants based on a low efficiency primer pair (PP56) and a high efficiency primer pair (PP22; Table S4). All the adults and infants fecal microbiota datasets where then processed using the same bioinformatic pipeline. In detail, they were quality filtered (Table S4) and analyzed using the same custom script based on the QIIME2 software suite [12] in order to avoid biases caused by different bioinformatic pipelines. Quality control retained sequences with a length between 140 and 400 bp and a mean sequence quality score >20, while sequences with homopolymers >7 bp and mismatched primers were omitted. 16S rRNA gene-based operational taxonomic units (OTUs) were defined at 100% sequence homology using DADA2 [13] and OTUs, i.e., cluster of identical sequences, constituted by only one sequence were removed. All reads were classified to the lowest possible taxonomic rank using QIIME2 [12,14] and a reference dataset from the SILVA database v.132 [15].

### 2.4. Statistical Analysis

The efficiency of the selected primer pairs on the core-microbiota of humans observed in a previous study [16] was used to perform a hierarchical clustering (HCL) analysis. In detail, the HCL analysis was performed by TM4 MeV software [17] and the cladogram was visualized through FigTree software (http://tree.bio.ed.ac.uk/software/figtree/).

## 3. Results and Discussion

### 3.1. Selection of Publicly Available 16S rRNA Gene-Based Primer Pairs

A wide range of (proposed) universal PCR primer pairs for amplification of a particular hypervariable region of the 16S rRNA gene have been designed for achieving accurate metagenomic profiling [8,18,19]. Nevertheless, due to their variable amplification performances, being caused by inconsistent sequence complementarity with corresponding bacterial 16S rRNA sequences, and variable discriminatory power of the amplified hypervariable region, no single PCR primer pair has become the gold standard for 16S rRNA gene-based microbial profiling. Nonetheless, 16S rRNA gene sequence databases have expanded and novel bioinformatic tools for PCR primer design have become available [7], although many studies still employ protocols that encompass the use of a suboptimal PCR primer pair. For this reason, in order to perform a comprehensive comparison of currently available primer pairs for 16S rRNA profiling, we performed an in-depth literature search for PCR primer pairs employed for 16S rRNA gene amplification and microbiota profiling (Figure S1 and Table S1). Furthermore, we expanded our database through the online resource probeBase, which encompasses 108 PCR primers described to be universal for bacteria. This effort allowed the identification of a total of 75 primer pairs that represent all current primers designed and used to amplify portions of the 16S rRNA gene.

### 3.2. In Silico Evaluation of the PCR Primer Efficiency

Metagenomic investigations based on the 16S rRNA gene allow a rapid and cost-effective view of the bacterial community present in a given environmental sample. Nevertheless, this methodology includes several critical steps that can introduce biases, such as storage of the samples and DNA extraction, that have already been described previously [20,21]. In this context, the selection of an efficient and accurate set of PCR primers targeting the 16S rRNA gene able to amplify all (or at least the vast majority of) known bacterial taxa plays a key role in obtaining complete and exhaustive bacterial profiles [8]. In order to evaluate the efficiency of the 75 PCR primer pairs included in this study for the amplification of the 16S rRNA gene, we performed an in silico PCR through the web-tool TestPrime 1.0. Notably, three mismatches were allowed in order to simulate high stringency PCR conditions [11]. Evaluation of the bacterial amplification capabilities (Table S2) revealed that 36, 8, and 32 primer pairs possess a calculated amplification efficiency of >90%, between 50 to 90%, and <50%, respectively. Curiously, six PCR primer pairs, i.e., PP4 (8F/907R), PP20 (8f, 616V/630R), PP55 (527F/1406R), PP73 (1099F/1541R (pH)), PP74 (P699D/P1425), and PP75 (P1525R/P609R) were predicted to have an amplification efficiency of <5%, indicating a rather poor performance of these PCR primers. In order to confirm the low amplification efficiencies of these six PCR primer pairs, we performed an in silico PCR through the web-tool Primer-BLAST using the NCBI nucleotide collection (nr) database [22]. Interestingly, five of the six PCR primer pairs, i.e., PP20, PP73, PP74, PP75, and PP4, were predicted to be able to amplify the bacterial 16S rRNA gene, suggesting that these primers contain sequences close to the ends of the 16S rRNA gene sequence that had not been included in the RefRN SILVA Database. These results led us to exclude the PCR primer pairs PP20, PP73, PP74, PP75, and PP4 from subsequent analyzes, as we could not evaluate their amplification efficiency with a comparable methodology.

### 3.3. Efficiency of PCR Primer Pairs on Human Gut Microbiota

In recent years, the human gut microbiota has been studied in depth to evaluate possible correlations between microorganisms and host, revealing its key role in human health [23]. Therefore, we decided to focus our interest on the 54 bacterial taxa commonly found in the human gut microbiota, as reported in previous study [16]. The analysis predicted that the PCR primer pairs with the best amplification performance were PP39 (Probio_Uni/Probio_Rev), PP41 (341F/534R), and PP72 (970F/1050R), showing an amplification efficiency of 98.55%, 98.52%, and 98.52%, respectively (Table S2).

**Figure 1:**
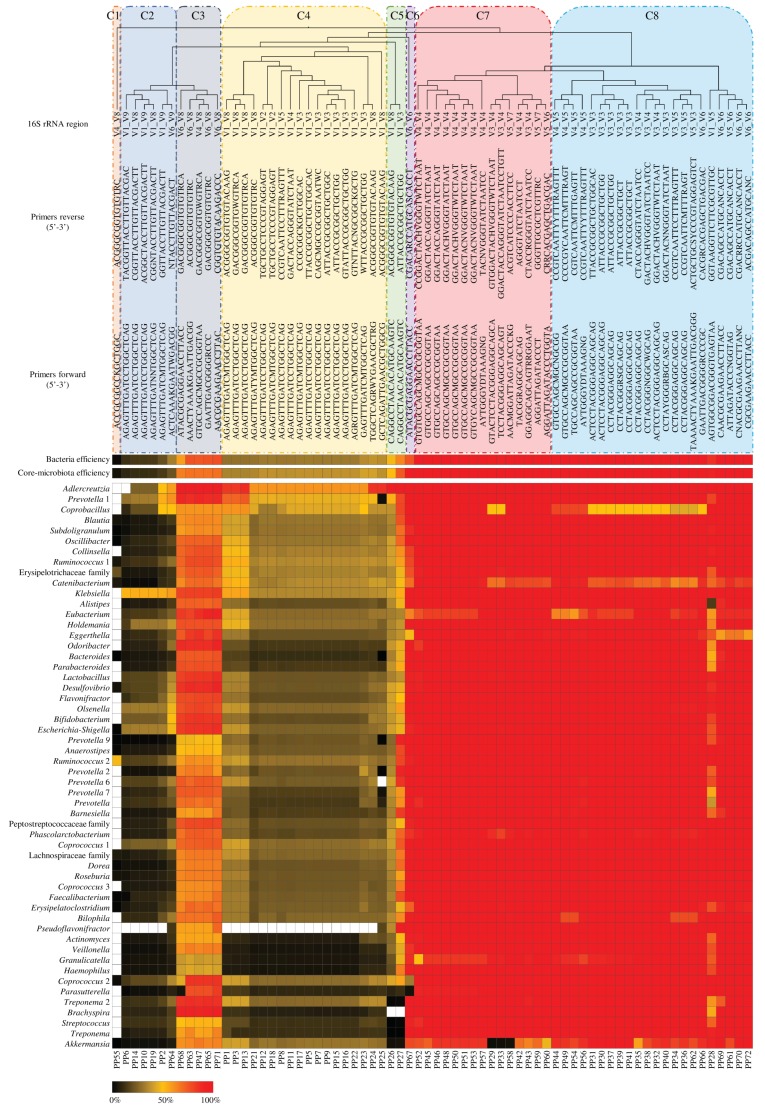
Amplification efficiency of primer pairs as calculated by web-tool TestPrime 1.0. A heat map showing primer pair amplification efficiency when targeting 54 bacterial taxa that are commonly found in the human gut microbiota. The PCR primer pair clusters were obtained by TM4 MeV software. The white cells indicate the inability of PCR primer pairs to amplify (members of) a bacterial genus.

In order to identify possible correlations between bacterial amplification capabilities and the PCR primer pairs, the efficiency of 54 selected bacterial taxa was used for a hierarchical clustering analysis, leading to the identification of eight different clusters (C; Figure 1), which strictly depend on the particular hypervariable region within the 16S rRNA gene that was targeted for amplification. In detail, clusters C1 and C2 included PCR primer pairs with a low predicted amplification efficiency of <30% (total average of 11.82% ± 5.26%), while clusters C3, C4, and C5 corresponded to sub-optimal efficiency of <70% (total average of 45.30% ± 13.99%). These PCR primer pairs were mainly represented by forward primers designed to amplify the V1 region and by reverse primers designed to target the V8 or V9 regions. Instead, clusters C6, C7, and C8 that were constructed to target the hypervariable V3, V4, V5, and V6 regions, were deduced to possess a high amplification efficiency of >90% (total average of 97.28% ± 1.12%). These results confirmed the notion that V3, V4, V5, and V6 are hypervariable regions that are highly suited for PCR-based 16S rRNA gene targeting [24,25]. However, the regions V4 and V5 are usually avoided for the detection of *Bifidobacterium* genus [5].

Focusing our interest on the bacterial genera, the C1, C2, and C4 clusters showed an inability to amplify *Pseudoflavonifractor*, while C1 and C5 presented an inability to amplify *Brachyspira* genus. Furthermore, C4 displayed higher efficiency (average of 76.69% ± 13.84%) for *Adlercreutzia* and *Prevotella* 1 genera compared to the other taxa (average of 35.16% ± 13.89%), indicating a probable over-estimation of these two bacterial genera. Interestingly, the C6, C7, and C8 clusters exhibited some issues in the amplification of the genus *Akkermansia* [26]. In detail, 14 PCR primer pairs out of 38 showed an efficiency of <90% and PP29 (336F/806R), PP33 (Bact340F/Bact806R), and PP58 (799F/1193R) were deduced to possess an amplification efficiency of <5% (average of 4.63% ± 0.81%), suggesting an underestimation of this bacterial genus.

### 3.4. Impact of Primer Performance on Metagenomics Results

**Figure 2:**
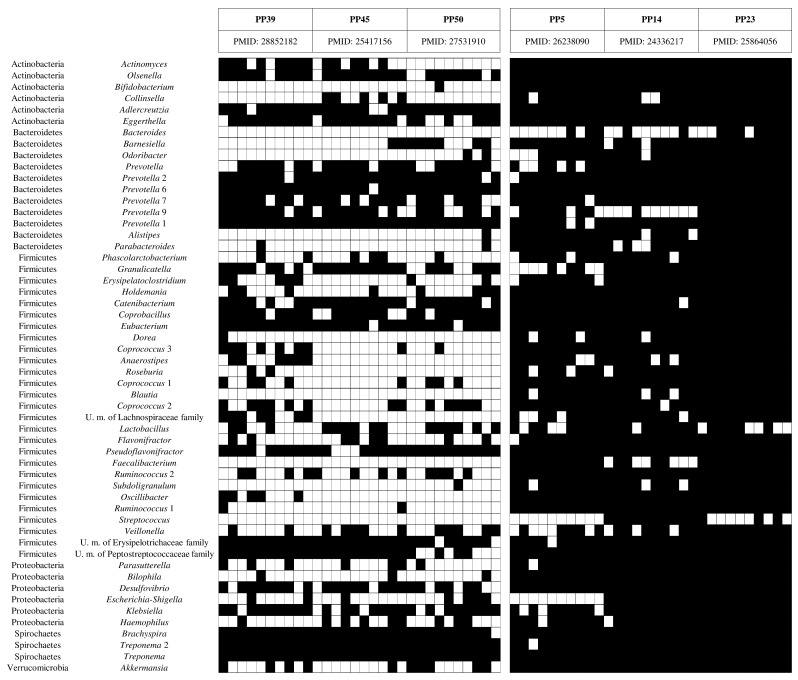
16S rRNA gene-based microbial profiling analysis of human fecal samples. The heat map reports the deduced relative abundance of 54 bacterial taxa that are commonly found in the human gut microbiota. The black cell indicated the absence of the bacterial genus.

In order to verify the impact of the predicted in silico primer efficiency on the taxonomic profiles reconstructed through 16S rRNA gene-based profiling analysis, we selected a total of 60 healthy human fecal samples belonging to six public metagenomic datasets (Table S4). Prior to performing a meta-analysis, all the 60 samples were re-analyzed using 10,000 reads per dataset and the same bioinformatics pipeline based on Qiime2 [12], DADA2 for OTU generation at 100% [13] identity, and the SILVA database v.132 [15]. In detail, we selected three studies based on primer pairs with an efficiency <50%, i.e., PP5, PP14, and PP23, and three studies that used primers pairs with a predicted amplification efficiency of >90%, i.e., PP39, PP45, and PP50. Notably, all the included datasets correspond to heathy human adults living in developed countries. The meta-analysis allowed the identification of a total of 200 bacterial genera with a relative abundance of >0.5% in at least one sample, which were used for further statistics (Table S5). Interestingly, taxonomical prediction revealed that the profiles obtained from PCR primer pairs with an amplification efficiency of >90% and <50% were able to detect 39.22% ± 4.05% and 9.40% ± 5.12% of the 200 observed bacterial taxa, respectively (Table S5). Focusing on the 54 bacterial taxa commonly found in the human gut microbiota reported in a previous study [16], the meta-analysis showed that samples based on PCR primer pairs PP39, PP45, and PP50 classify 30 ± 4 genera (54.88% ± 6.50%), while samples obtained from PP5, PP14, and PP23 identify only 5 ± 4 genera (8.95% ± 6.56%; Figure 2 and Table S5). These data were also confirmed by comparison of two data sets obtained from 10 and 9 fecal samples corresponding to infants profiled with the high-efficiency PCR primer pair PP56 and the low efficiency PCR primer pair PP22, respectively (Table S5). Intriguingly, PCR primer pair PP56 allowed the detection of a total of 26 ± 10 taxa with a relative abundance >0.5%, while PCR primer pair PP22 detected just 13 ± 1 genera. Although the different DNA extraction procedures employed in these two different studies may have impacted on the number of species detected, the remarkable differences in profiling accuracy observed between high and low performance primers set confirmed that the use of a validated and tested protocol of 16S rRNA profiling is fundamental to avoid underestimation of specific bacterial genera. Moreover, these results underline the critical importance to carefully consider the applied protocols when inspecting or comparing data published in previous studies.

### 3.5. In Silico Evaluation of the PCR Primer Efficiency of the Bifidobacterial Species

**Figure 3:**
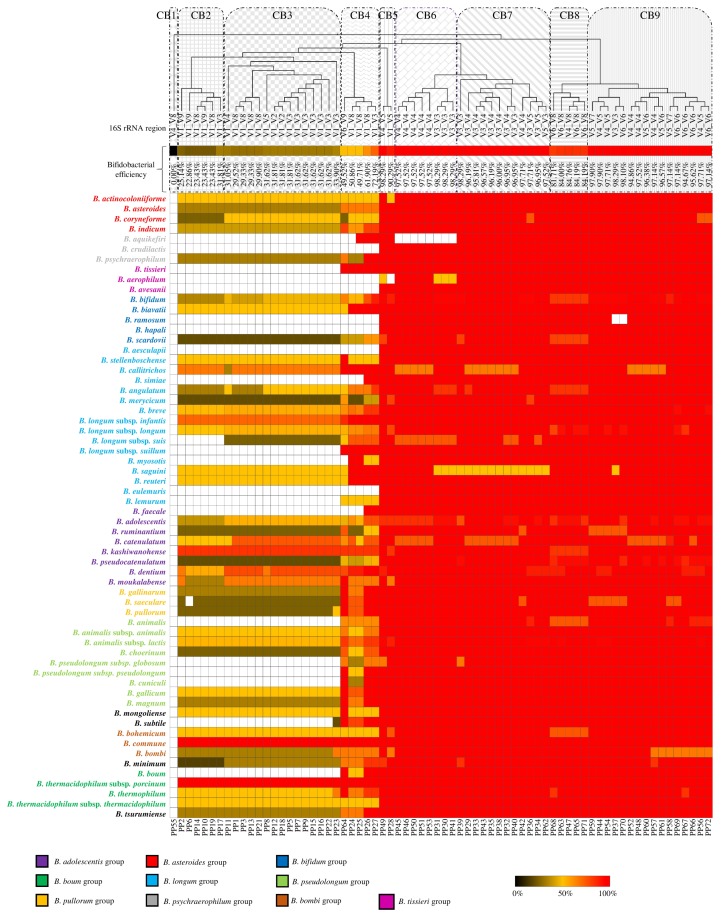
PCR primer pair amplification efficiency towards all so far known bifidobacterial species. A heat map illustrating PCR primer pair efficiency on all so far known bifidobacterial species. The bifidobacterial clusters were obtained by TM4 MeV software considering the primer pair efficiency. The inability of a given PCR primer pair to amplify a particular bifidobacterial species was highlighted by a white cell.

Our analyses revealed that several tested PCR primers were able to efficiently amplify only part of the overall number of expected bacterial genera. In this context, we focused our interest on the *Bifidobacterium* genus in order to explore intra-genus variabilities that may cause underrepresentation of the bacterial population. Notably, this taxon was chosen as a test case due to the high number of bifidobacterial sequences present in the SILVA database (525) covering the main bifidobacterial species that are currently known. In silico PCR, performed with the web-tool TestPrime, deduced that the 75 selected primers can be divided into nine clusters (CB; Figure 3 and Table S3). This analysis showed that CB1, i.e., PP55 (527f/1406r), was unable to amplify the *Bifidobacterium* genus, while CB2 and CB3 possessed an efficiency lower than 35%, thus causing a severe underestimation of the *Bifidobacterium* genus. Furthermore, CB4 displayed an average efficiency of 56.84% ± 10.02%, while C5 and C6 revealed an average efficiency of 95.46% ± 4.52%. Optimal primers for the amplification of the bifidobacterial species therefore seem to be PP30 (P338f/P518r), PP31 (338F/533R), PP37 (341F/518R), PP39 (Probio_Uni/Probio_Rev), PP41 (341F/534R), and PP49 (515F/909R) with an overall efficiency of 98.28%, although only PP39 and PP49 amplify all species tested (Figure 2), thereby fully covering currently known bifidobacteria.

The division of the bifidobacterial species in the 10 known phylogenetic groups [27] allowed to identify a possible correlation between primer pairs and species amplification. In detail, CB1, CB2, and CB3 clusters, which are mainly represented by forward PCR primers designed to amplify the V1 region and by reverse PCR primers designed to amplify V8 and V9 regions, showed an inability to amplify several species belonging to *B. psychraerophilum* group, *B. tissieri* group, *B. bifidum* group, *B. longum* group, and *B. pseudolongum* group. Curiously, the CB6 cluster, characterized by PCR primers pairs that amplify 16S rRNA gene regions spanning from nucleotide 515 to nucleotide 806 and from nucleotide 338 to nucleotide 534 [28] indicated an inability to amplify *B. aquikefiri*, thus highlighting the importance of selection of optimal 16S rRNA gene primer pairs.

## 4. Conclusions

The 16S rRNA gene is one of the main gene markers used to profile bacterial communities. The selection of specific PCR primer pairs is essential for assessing the microbiota composition. The in silico PCR performed in this study allowed us to identify marked differences of the amplification efficiency of the 75 PCR primer pairs tested. In detail, we found that the most efficient hypervariable regions for phylogenetic and taxonomic analysis were V3, V4, V5, and V6. In particular, PCR primer pairs PP39 (Probio_Uni/Probio_Rev), PP41 (341F/534R), and PP72 (970F/1050R) showed the highest amplification efficiency of 54 bacterial taxa that are commonly present in the human gut microbiota. Interestingly, when we focused on the bifidobacterial species the PCR primer pair PP55 (527f/1406r) was deduced to be incapable of amplifying the targeted region of the 16S rRNA gene of any member of this bacterial genus, emphasizing the importance of selection of optimal 16S rRNA gene primer pairs so as to avoid underestimating bacterial genera. Thus, the data here described might be of pivotal importance in order to identify the most appropriated primers/experimental conditions for the profiling of the human gut microbiota.

## Supplementary Materials

The following are available online at https://www.mdpi.com/2076-2607/8/1/131/s1, **Figure S1.** Schematic depiction of the approaches used in this study; **Table S1.** List of primer pairs included in the study; **Table S2.** A heat map illustrating the PCR primer pair amplification efficiency on 54 bacterial taxa commonly found in the human gut microbiota. **Table S3.** A heat map reporting the PCR primer pair amplification efficiency on all so far described bifidobacterial species; **Table S4.** List of public data sets included in the meta-analysis; **Table S5.** Taxonomic prediction of the 79 samples included in this study through 16S rRNA gene-based microbial profiling.
